# Impact of Organic and Conventional Systems of Coffee Farming on Soil Properties and Culturable Microbial Diversity

**DOI:** 10.1155/2016/3604026

**Published:** 2016-03-03

**Authors:** Kulandaivelu Velmourougane

**Affiliations:** Post Harvest Technology Lab, Coffee Research Sub Station, Coffee Board, Chettalli, Coorg, Karnataka 571 248, India

## Abstract

A study was undertaken with an objective of evaluating the long-term impacts of organic (ORG) and conventional (CON) methods of coffee farming on soil physical, chemical, biological, and microbial diversity. Electrical conductivity and bulk density were found to increase by 34% and 21%, respectively, in CON compared to ORG system, while water holding capacity was found decreased in both the systems. Significant increase in organic carbon was observed in ORG system. Major nutrients, nitrogen and potassium, levels showed inclination in both ORG and CON system, but the trend was much more pronounced in CON system. Phosphorus was found to increase in both ORG and CON system, but its availability was found to be more with CON system. In biological attributes, higher soil respiration and fluorescein diacetate activity were recorded in ORG system compared to CON system. Higher soil urease activity was observed in CON system, while dehydrogenase activity does not show significant differences between ORG and CON systems. ORG system was found to have higher macrofauna (31.4%), microbial population (34%), and microbial diversity indices compared to CON system. From the present study, it is accomplished that coffee soil under long-term ORG system has better soil properties compared to CON system.

## 1. Introduction

Organic farming has been defined as an agricultural production system that avoids or excludes the use of synthetic fertilizers and pesticides. Organic farming relies completely on crop rotations, use of animal and green manure, and biological pest control in order to maintain the soil productivity, supply of plant nutrients, and control/management of insects, weeds, and other pests [1]. Research work pertaining to long-term environmental impacts of organic and conventional production systems has focused primarily on indicators related to soil quality [2, 3].

Coffee is one of the most important plantation crops grown in India and is a major foreign exchange earning commodity. Coffee is predominantly grown at high altitudes/hilly regions of southern states of India where a tropical climate prevails accompanied by well drained soils rich in humus. Coffee plantation industry not only plays an important role in preservation of ecosystem in the tropical forest hills of Western and Eastern Ghats, but also provides employment opportunities to nearly 500,000 local residents.

Of late, production and consumption of organic coffee have gained paramount importance among the coffee importing countries and organic coffee consumption all over the world is around 4% [4]. To our knowledge, no comprehensive studies related to impacts of farming systems on soil properties in coffee have been reported from India. Based on the above background, the present study was undertaken with an objective of evaluating the long-term impact of organic and conventional methods of coffee production system on soil physical, chemical, biological, and microbial diversity.

## 2. Materials and Methods

### 2.1. Description of Field and Experimental Setup

The experimental sites (arabica coffee,* Coffea arabica* L.) for the study were selected in Coffee Research Farm, Chettalli, Kodagu District, Karnataka, India, which is situated 989 m above the mean sea level and gets an annual rainfall of approximately 1700 mm and is characterized as having red lateritic soils under tropical evergreen climate. The experimental plots (6 acres each in randomized block design) have been maintained under organic and conventional system for the last 12 years. The initial soil parameters were obtained from the previously conducted study at this station. The soil pH (1 : 2 water) was 5.87, electrical conductivity (EC) 0.152 dSm^−1^, bulk density (BD) 1.02 g/cm^3^, water holding capacity (WHC) 57.8%, organic carbon (OC) 1.73%, available nitrogen (N) 365 kg/ha, available phosphorus (P) 27.5 kg/ha, and available potassium (K) 360 kg/ha. The experimental plots (organic and conventional) were wholly covered by a single dominant arabica variety (Selection 795) in India with spacing of 0.3 × 0.3 m. CON coffee plots received a blanket nutrient schedule of 40 : 30 : 40 (N : P_2_O_5_ : K_2_O kg ha^−1^ per year) and nitrogen was applied in three splits. Leaf rust was managed with 0.5% Bordeaux mixture and appropriate chemical application was adopted for white stem borer control in CON system, while ORG coffee plots were amended with 5 tonnes of organic manure (farmyard manure and compost) per acre once in the cropping cycle. Weed control in conventional system was managed by one herbicide spraying and two manual weeding rounds, while ORG plots were managed with three rounds of manual weeding. Pest and disease problem in organic system were managed using neem based formulation for sucking pests, lime swabbing, pheromone traps, and Broca traps for white stem borer, and 0.5% Bordeaux spray for rust control. Other crop management operations like pruning, harvesting, and processing were similar for both the systems.

### 2.2. Soil Physicochemical Analysis

The experimental soil samples (5 nos./block) for analysis were collected randomly at a depth of 0–15 and 15–30 cm at flowering, air-dried, crushed, and sieved through a 2 mm mesh screen and used for further analysis. Water holding capacity (WHC) was estimated by Keen-Raczkowski method as outlined by Piper [5] while the determination of pH (1 : 2) was done by a digital pH meter (ELICO-L11 62). The soil organic carbon (SOC) and available nitrogen (N) were estimated by Walkley and Black rapid titration method [6] and Kjeldahl method, respectively [7]. Available phosphorus (P) and available potassium (K) were estimated following standard methods [8–10].

### 2.3. Soil Biological Analysis

The selected soil biological parameters, namely, soil respiration, soil dehydrogenase activity (DHA), soil urease activity, and fluorescein diacetate hydrolysis (FDA), were performed on the freshly collected samples following standard methods. The abovementioned variables were selected as they are used as soil quality/health indicators. Soil respiration was measured as the CO_2_ evolved from moist soil, adjusted to 55% water holding capacity, and preincubated for three days at 22–25°C with 10 mL of 1 M NaOH. The CO_2_ production was then measured by back titrating unreacted alkali with 1 N HCl to determine CO_2_-C [11]. Dehydrogenase activity (DHA) was determined following the method of Casida [12] by the reduction of 2,3,5-triphenyl tetrazolium chloride (TTC). Soil (10 g) was incubated for 24 h with TTC at 27°C in duplicate. The triphenyl formazan (TPF) formed was extracted with acetone and measured spectrophotometrically at 546 nm. Dehydrogenase activity was expressed as *µ*g TPF g^−1^ dry soil h^−1^. Urease activity was assayed in duplicate by the method described by Tabatabai and Bremner [13], which involves the determination of the ammonium released by urease activity when 5 g of soil is incubated with 9 mL of 0.05 M tris(hydroxymethyl)aminomethane (THAM) buffer (pH 9.0), 1 mL of 0.2 M of urea solution and toluene at 37°C for 2 h. The ammonium released was determined by a procedure involving treatment of the incubated soil sample with 2.5 M KC1 containing a urease inhibitor (Ag_2_SO_4_) and steam distillation of an aliquot of the resulting soil suspension with MgO for 4 min. Urease activity was expressed as *µ*g NH_4_-N g^−1^ dry soil. FDA was measured following the method of Schnürer and Rosswall [14] using 3,6-diacetyl fluorescein as substrate and measuring the fluorescence at 490 nm. The soil micro- and macrofauna population was analyzed (0–30 cm depth) using Berlese funnel method [15].

### 2.4. Soil Microbiological and Diversity Analysis

Soil samples collected at 0–15 cm and 15–30 cm depth from ORG and CON plots were serially diluted in 90 mL Ringer's solution up to 10^−4^ dilution and 1 mL of aliquot was pour plated into selective media (Nutrient Agar for bacteria, Martin's Rose Bengal Agar for fungi, Ken Knights and Munaier's Agar for actinomycetes, and Buffered Yeast Agar for yeast). The plates were incubated at optimum temperature (25°C ± 1°C) in triplicate. The functional/physiological groups of microbes were enumerated by following standard microbiological methods [16]. The functional groups from the soil samples were enumerated using Pikovskaya Agar for phosphorus solubilizing microbes (PSM), Waksman Number 77 media for* Azotobacter*, and King's B media for fluorescent pseudomonads. The microbial colonies appearing after the stipulated time period of incubation (3 days for bacteria and yeast; 5 days for fungi; 7 days for actinomycetes) were counted and expressed as colony forming units (CFUs)/g of the sample. The culturable microbial diversity indices for ORG and CON systems were determined following standard methods [17].

### 2.5. Statistical Analysis

Significant (*p* < 0.01 and *p* < 0.05) differences between ORG and CON method of coffee farming on soil attributes were analyzed using SPSS (version 7.5) software. Tukey multiple comparison tests were done to determine the differences between ORG and CON method of coffee farming.

## 3. Results and Discussion

### 3.1. Effect of Organic and Conventional Method of Coffee Farming on Soil Physicochemical Properties

**Figure 1:**
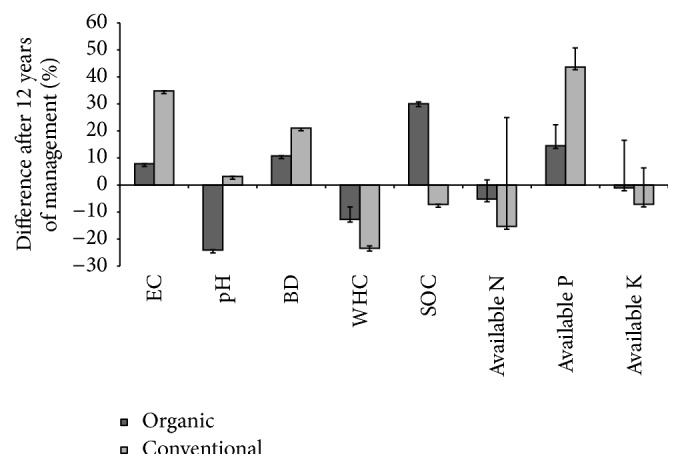
Impact of 12 years of organic and conventional management in coffee on selected soil physical and chemical properties. EC: electrical conductivity; BD: bulk density; WHC: water holding capacity; SOC: soil organic carbon; Av.N: available nitrogen; Av.P: available phosphorus; Av.K: available potassium.

In soil physical properties, significant difference was observed in electrical conductivity (EC) between organic (ORG) and conventional (CON) systems at both the soil depths, while bulk density (BD) showed significance only at 0–15 cm for both the systems (Table 1). Water holding capacity (WHC) of the soil at 0–15 cm of ORG system was found to be significantly higher by 53.36% compared to CON system (45%), while WHC of ORG and CON system at 15–30 cm depth was found to be nonsignificant. In chemical properties, soil organic carbon (SOC), available phosphorus (P), and potassium (K) showed statistical significance at both the depths; available nitrogen (N) showed significance only at 0–15 cm. From the mean analysis of soil physical and chemical properties after 12 years of management, it was found that EC in CON system increases by 36% while it rises to 6.9% in ORG system (Figure 1). pH was found to decrease by 24% in ORG system, while its value rose by 3.6% in CON system. BD was found to increase in both ORG (12.7%) and CON (22.5%) system, while WHC was found to decrease in both ORG (10.8%) and CON (19.8%). Significant increase in SOC of ORG system (15.6%) was found compared to CON system, where the SOC was found to decrease by 16.7%. In major nutrients, N and K were found to show inclination in both ORG (5.2% and 0.97%) and CON (15.7% and 4.3%), respectively, for 0–15 and 15–30 cm soil depth, but the trend was much more pronounced in CON system. P was found to increase in both ORG (10.9%) and CON (41.8%) system, but its availability was found to be more with CON system.

From the study, it is understandable that organic farming has better advantage over the conventional farming though many of the soil properties do not show statistical significant results. In physical properties, the lesser BD in ORG system indicates better status of soil structure. Bulk density is an indicator of soil compaction and it reflects the soil's ability to function for structural support, water and solute movement, and soil aeration. Though the BD value has slight increase (1.1 g/cm^3^) from the initial values (1.02 g/cm^3^), application of organic manure in ORG system was found to record lesser BD values compared to CON system (1.24 g/cm^3^). Soils amended with high organic manures are reported to have lesser BD [18]. The increased WHC of ORG system is also attributed to the higher availability of organic matter in the soil compared to CON management. The increase in soil EC in CON system compared to ORG system is a clear indication of salts accumulation from the fertilizer usage. However, the drop in the pH in ORG system compared to CON system indicates the organic manure effect on soil reaction. Continuous application of organic manures (cow dung,* Leucaena* leaves, farm residues, and* Sesbania*) in tropical soils has been shown to increase the organic carbon content of soil [19]. Continuous application of organic manure is also reported to increase soil enzyme and microbial activities [20]. Higher soil nutrient status in coffee under organic cultivation was also reported [21]. Interestingly, higher available P was recorded in CON farming compared to ORG; the increase of P content in CON system may be mainly due to application of higher P fertilizers in CON system compared to ORG farms.

### 3.2. Effect of Organic and Conventional Method of Coffee Farming on Soil Biological Attributes

Significant and higher soil urease and FDA activity was recorded in ORG production system compared to CON system at both the depths, while soil respiration found record significant difference only at 0–15 cm (Table 2). DHA activity was not found to show significant difference between ORG and CON production system at both the depths. From the mean analysis of soil biological properties after 12 years of management, it was found that soil respiration increases by 15.4 and 8.6%, respectively, for 0–15 and 15–30 cm in ORG system compared to CON system. In contrast, urease activity was found to be higher in CON system (34 and 36% for 0–15 and 15–30 cm, resp.) compared to ORG system. Though DHA does not produce significant differences between ORG and CON systems, its activity was higher in ORG system (16 and 15% for 0–15 and 15–30 cm, resp.). FDA activity was observed to be higher by 25 and 31% for 0–15 and 15–30 cm, respectively, in ORG system compared to CON system.

The higher soil respiratory activity in ORG system indicates the soil health promoting functions of organic farming on microbial activity. The higher FDA activity in organic coffee cultivation indicates higher microbial activity compared to conventional method of coffee cultivation. The higher soil urease and dehydrogenase activity in ORG management shows the effectiveness of microbial activity in that system compared to CON system. Enzymes are important soil components involved in the dynamics of soil nutrient transformations. Enzyme activity in the soil environment is considered to be a major contributor of overall soil microbial activity and soil quality [22]. Urease is an important enzyme in soil mediating the conversion of organic nitrogen to inorganic nitrogen by the hydrolysis of urea to ammonia. Increase in soil urease activity with increasing organic matter content has been already reported [13].

### 3.3. Effect of Organic and Conventional Method of Coffee Farming on Soil Micro- and Macrofauna

ORG system recorded significantly (*p* < 0.01) higher population of Oribatid mites (*Pelops*), Thrips, and scarabaeid beetle compared to CON coffee system (Table 3), whereas the population of Oribatid mites (*Eulohmannia*), Symphylans, and springtails was found to show significance at 5% in ORG system. No significant differences were found in the population of proturans, Japygids, rove beetles, Millipedes, and Centipedes between ORG and CON coffee production system. From the mean analysis of micro- and macrofauna population at 0–30 cm of soil depth after 12 years of management, the population of* Eulohmannia*,* Pelops*, proturans, Thrips, Symphylans, rove beetles, springtails, and scarabaeid beetles was found to increase under ORG system compared to CON system by 50, 44.8, 35.5, 53.6, 62.7, 12, 38, and 72%, respectively. Interestingly, the population of Japygids, pauropods, pseudoscorpions, Millipedes, and Centipedes was found to increase under CON system compared to ORG system by 4.5, 43, 250, 61, and 59%, respectively. Overall, the ORG system recorded higher (31.4%) total micro- and macrofauna population compared to CON system.

Most of the earlier studies reported enhancement in faunal biodiversity in organic farms compared to conventional farms in most studies [23]. Many studies continue to support a positive association between organic management and on-farm biodiversity for predatory arthropods [24]. Oehl et al. [25] found a greater diversity of soil microorganisms on organic farms than on conventional farms. In the present study, the higher micro- and macrofauna and microflora population in ORG farming clearly indicates the management impact of safer methods of control of insects pests in coffee (pruning, lime swabbing, neem formulation, borer tracing, and pheromone traps) compared to chemical control methods in CON farming, wherein most of the harmful plant protection chemicals, namely, lindane (control of white stem borer in coffee) and endosulfan (coffee berry borer control), are used. The high occurrence of micro- and macrofauna and microbial population in organic coffee system is an indication of positive effects of employing organic farming in coffee farms. These results are similar to the research findings by Fraser et al. [26].

### 3.4. Effect of Organic and Conventional Method of Coffee Farming on Culturable Microbial Population and Microbial Diversity Indices

Higher culturable microbial population was recorded in surface soil (0–15 cm) compared to 15–30 cm soil depth (Table 4). The total culturable microbial population was found to be significantly (*p* < 0.01) higher in ORG system compared to CON coffee system. In microbial groups, bacterial population was found to be significantly (*p* < 0.05) higher in ORG system compared to CON system, whereas no significant differences in bacterial population between ORG and CON system were found. Nonsignificant differences in population of fungi and actinomycetes between ORG and CON systems were found at both the soil depths, whereas the yeast population was found to be significantly higher in ORG system at 0–15 cm (*p* < 0.01) and 15–30 cm (*p* < 0.05), respectively. In functional microflora, the population of PSM was found to be significantly (*p* < 0.05) higher in ORG system compared to CON system, while the population of* Azotobacter* spp. was found to yield nonsignificant differences between ORG and CON systems. Significantly (*p* < 0.05) higher* P*.* fluorescens *was observed in ORG system at 0–15 cm, while 15–30 cm soil depth recorded nonsignificant differences between ORG and CON systems. The mean analysis of culturable population after 12 years of management revealed that the populations of bacteria, fungi, and yeasts were found to be higher in ORG system compared to CON system by 24 and 6.7% at 0–15 and 15–30 cm soil depth, 12 and 48%, and 59 and 32%, respectively. Actinomycetes population was found to be higher (32.6%) in ORG system at 0–15 cm, while CON system recorded higher actinomycetes population (19.3%) at 15–30 cm soil depth. In functional microflora, PSM,* Azotobacter* spp., and* P*.* fluorescens* were found to increase by 44 and 49, 13 and 17, and 29 and 17% in ORG system compared to CON system at 0–15 and 15–30 cm, respectively. The overall higher value of total culturable microbial population in ORG compared to CON system was found to be 34% and 15% for 0–15 and 15–30 cm, respectively.

In general, the ORG system recorded higher microbial diversity indices compared to CON system at both the soil depths (Table 5). In ORG system, Shannon-Weiner Index (*H*′), Simpson's Reciprocal Index (1/*D*), and Shannon Evenness (*E*) recorded significantly (*p* < 0.05) higher value compared to CON system at 0–15 cm soil depth, while, at 0–30 cm soil depth, none of the diversity indices produced significant results.

The increased microbial activity and diversity in the surface soils are attributed to the greater availability of organic carbon, nutrients, moisture, and aeration status compared to subsurface. Depth of root penetration and nutrient exhaustive characteristics of crops also may be another reason for the decline of culturable microbial population in deeper layers. Impact of soil depth on proportions of microbial activity has been already reported [27]. Organic practices were found to rapidly improve soil microbial characteristics and slowly increase soil organic C [2]. Organic manuring with plant residues was reported to have a stronger impact on soil microbial activity as compared to other fertilization methods [28]. The impacts of chemical fertilization on growth and activity of microorganisms are often reported to be species specific [29, 30]. Velmourougane et al. [31, 32] reported higher soil biological activity in coffee grown under organic management in coffee growing regions of India.

## 4. Conclusions

From the present study, it was evident that adopting organic method of cultivation can help to build and improve the soil fertility in terms of physical, chemical, biological, and microbiological diversity in coffee farms compared to conventional method of coffee farming at both surface and subsurface soil.

## Figures and Tables

**Table 1:** Organic and conventional method of coffee farming on soil physical and chemical properties.

Management systems	EC (dSm^−1^)		pH (1 : 2)		BD (g/cm^3^)		WHC (%)		Soil organic carbon (%)		Available N (kg/ha)		Available P (kg/ha)		Available K (kg/ha)	
	0–15	15–30	0–15	15–30	0–15	15–30	0–15	15–30	0–15	15–30	0–15	15–30	0–15	15–30	0–15	15–30
Organic	0.162^b^	0.163^b^	4.37^b^	4.54^b^	1.10^b^	1.20^a^	54.8^a^	48.3^a^	2.50^a^	2.00^a^	351^a^	341^a^	36^b^	25^b^	376^a^	351^a^
Conventional	0.211^a^	0.203^a^	6.05^a^	6.12^a^	1.24^a^	1.26^a^	47.0^b^	45.7^a^	1.50^b^	1.38^b^	279^b^	336^a^	44^a^	34^a^	354^b^	335^b^
SEm	0.025	0.020	0.84	0.79	0.07	0.03	3.9	1.3	0.50	0.31	36.3	2.4	4.03	4.42	11.0	7.9
CD (0.05)	0.003	0.014	0.116	0.041	0.04	0.045	2.87	NS	0.146	0.146	12.34	NS	4.68	2.54	8.57	7.78
CD (0.01)	0.005	0.022	0.182	0.065	0.062	NS	4.51	NS	0.229	0.245	19.35	NS	7.34	3.98	13.45	12.21

All the values are mean of 5 replications.

EC: electrical conductivity; BD: bulk density; WHC: water holding capacity.

SEm: standard error of mean; CD: critical difference; NS: nonsignificant.

Superscript letters indicate statistical significance following Tukey multiple comparison tests.

**Table 2:** Organic and conventional method of coffee farming on selected soil biological properties.

	Soil respiration		Urease		DHA		FDA	
Management systems	(CO_2_ mg/50 g)		(*µ*g NH_4_-N/g^−1^ h^−1^)		(*µ*g TPF g^−1^ 24 h^−1^)		(fluorescein *µ*g/g^−1^)	
	0–15	15–30	0–15	15–30	0–15	15–30	0–15	15–30
Organic	29.3^a^	18.5^a^	36.8^b^	27.8^b^	12.3^a^	9.2^a^	46.0^a^	32.0^a^
Conventional	24.8^b^	16.9^a^	49.3^a^	37.8^a^	10.3^a^	7.8^a^	34.3^b^	22.1^b^
SEm	2.2	0.8	6.2	5.0	1.0	0.7	5.9	5.0
CD (0.05)	4.41	NS	4.47	3.42	NS	NS	5.54	2.63
CD (0.01)	NS	NS	7.01	5.37	NS	NS	8.69	4.13

All the values are mean of 5 replications.

SEm: standard error of mean; CD: critical difference; NS: nonsignificant.

DHA: dehydrogenase activity; FDA: fluorescein diacetate activity.

Superscript letters indicate statistical significance following Tukey multiple comparison tests.

**Table 3:** Organic and conventional method of coffee farming on selected soil micro and macrofauna population.

Management systems	Macrofauna population (nos./sq.m at 0–30 cm soil depth)													
	Oribatid mites (*Eulohmannia*)	Oribatid mites (*Pelops*)	Proturans	Japygids	Thrips	Symphylans	Pauropods	Rove beetles	Springtails	Pseudoscorpions	Millipedes	Centipedes	Scarabaeid beetle larvae/grub	Total population
Organic	17.6^a^	20.3^a^	6.2^a^	2.2^a^	14.0^a^	7.5^a^	3.5^b^	2.5^a^	25.8^a^	3.0^b^	2.3^a^	1.7^a^	6.5^a^	113^a^
Conventional	8.8^b^	11.2^b^	4.0^a^	2.3^a^	6.5^b^	2.8^b^	5.0^a^	2.2^a^	16.0^b^	10.5^a^	3.7^a^	2.7^a^	1.8^b^	77.5^b^
SEm	1.6	1.6	0.7	0.3	1.2	0.9	0.4	0.3	2.4	1.2	0.4	0.3	0.8	17.7
CD (0.05)	6.13	2.33	NS	NS	2.71	3.73	1.1	NS	9.18	2.63	NS	NS	2.16	13.2
CD (0.01)	NS	3.66	NS	NS	4.26	NS	NS	NS	NS	4.13	NS	NS	3.4	20.7

All the values are mean of 5 replications.

SEm: standard error of mean; CD: critical difference; NS: nonsignificant.

Superscript letters indicate statistical significance following Tukey multiple comparison tests.

**Table 4:** Organic and conventional method of coffee farming on culturable microbial population.

	Microflora population (CFU × 10^4^/g) at 0–15 and 15–30 cm soil depth															
Management systems	Bacteria		Fungi		Yeast		Actinomycetes		PSM		*Azotobacter *spp.		*P. fluorescens *		Total population	
	0–15	15–30	0–15	15–30	0–15	15–30	0–15	15–30	0–15	15–30	0–15	15–30	0–15	15–30	0–15	15–30
Organic	76.3^a^	43.5	7.5	5.0	44.1^a^	19.1^a^	21.5	8.8	4.5^a^	3.5^a^	2.3	1.8	26.5^a^	13.3	182.6^a^	95.1^a^
Conventional	58.1^b^	40.6	6.6	2.6	18.0^b^	13.0^b^	14.5	10.5	2.5^b^	1.8^b^	2.0	1.5	18.8^b^	11.1	121.0^b^	81.0^b^
SEm	9.1	1.45	0.45	1.2	13.1	3.05	3.5	0.85	1.0	0.85	0.15	0.16	3.85	1.10	30.8	7.1
CD (0.05)	12.77	NS	NS	NS	5.35	4.92	NS	NS	1.99	1.58	NS	NS	5.25	NS	22.1	9.2
CD (0.01)	NS	NS	NS	NS	8.39	NS	NS	NS	NS	NS	NS	NS	NS	NS	31.2	12.9

All the values are mean of 5 replications.

SEm: standard error of mean; CD: critical difference; NS: nonsignificant; CFU: colony forming unit; *P. fluorescens*: *Pseudomonas fluorescens*.

Superscript letters indicate statistical significance following Tukey multiple comparison tests.

**Table 5:** Organic and conventional method of coffee farming on microbial diversity indices.

Systems	Soil depth (cm)	Microbial diversity indices				
		Shannon-Weiner Index (*H*′)	Simpson's Index of Diversity (*D*)	Simpson's Reciprocal Index (1/*D*)	Shannon Evenness (*E*)	Simpson's Evenness (*E*)
Organic	0–15	2.68^a^	0.40	2.52^a^	0.90^a^	0.55
Conventional	0–15	2.56^b^	0.44	2.28^b^	0.85^b^	0.51
	CD (0.05)	0.035	NS	0.02	0.024	NS

Organic	0–30	2.54	0.42	2.32	0.84	0.51
Conventional	0–30	2.55	0.43	2.27	0.85	0.51
	CD (0.05)	NS	NS	NS	NS	NS

All the values are mean of 5 replications; CD: critical difference; NS: nonsignificant.

Superscript letters indicate statistical significance following Tukey multiple comparison tests.
